# MicroRNA-34-5p regulates the expression of ecdysteroid receptor (ECR) in the process of salivary gland degeneration of ticks

**DOI:** 10.1186/s13071-025-06842-8

**Published:** 2025-05-23

**Authors:** Shanming Hu, Yanan Wang, Yongzhi Zhou, Jie Cao, Houshuang Zhang, Jinlin Zhou

**Affiliations:** Key Laboratory of Animal Parasitology of Ministry of Agriculture, Shanghai Veterinary Research Institute, Chinese Academy of Agricultural Sciences, Shanghai, 200241 China

**Keywords:** Salivary gland degeneration, Apoptosis, miR-34-5p, RhECR

## Abstract

**Background:**

The salivary glands of female ticks rapidly degenerate after feeding via programmed cell death mediated by an ecdysteroid receptor (ECR). The degeneration includes both apoptosis and autophagy. The process of degeneration can also be regulated by microRNAs (miRNAs), but the underlying mechanism of miRNA involvement in salivary gland degeneration remains incompletely understood. Here, we demonstrate that microRNA34-5p (miR-34-5p) regulates the process of salivary gland degeneration in *Rhipicephalus haemaphysaloides* by modulating the target gene RhECR.

**Methods:**

Dual luciferase reporter assays and phenotypic rescue experiments identified RhECR as a direct target of miR-34-5p. The overexpression and inhibition of miR-34-5p were quantified by hematoxylin and eosin (H&E) and Terminal deoxynucleotidyl transferase dUTP Nick-End Labeling (TUNEL) staining.

**Results:**

The results showed that miR-34-5p inhibited the expression of RhECR to retard apoptosis in salivary gland acini. The study identified the roles of miR-34-5p and RhECR and their interactions in tick salivary gland degeneration.

**Conclusions:**

The findings will aid in the application of ECR genes for tick control.

**Graphical Abstract:**

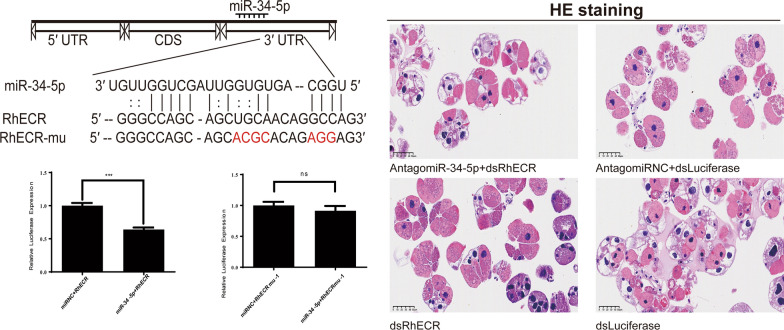

**Supplementary Information:**

The online version contains supplementary material available at 10.1186/s13071-025-06842-8.

## Background

Ticks are obligatory blood-feeding arthropods that act as vectors for many pathogens [1]. Ticks often stay attached to their hosts and feed for several days or even weeks [2]. The salivary glands of ticks mediate diverse functions that ensure tick survival [3]. Tick-borne pathogens (TBP) are transmitted to the host via the saliva during feeding [4].

The salivary glands of female ticks rapidly degenerate within 4 days after engorgement [5]. Salivary gland degeneration begins at the rapid phase of feeding, and it may be caused by apoptosis and autophagy related to the increase of ecdysone in the hemolymph [6, 7]. During degeneration, the granular acini undergo DNA fragmentation, and the activity of the caspase enzyme increases [8, 9]. According to a comprehensive analysis of global protein changes, proteins associated with apoptosis and autophagy were altered in expression, with enzymes linked to the degradation of DNA and proteins being consistently upregulated [10]. In *Rhipicephalus haemaphysaloides*, a recent study identified three caspases (RhCaspases7, RhCaspases8, and RhCaspases9) involved in the degeneration of tick salivary glands [11]. Recent research has demonstrated that silencing the ECR inhibits salivary gland degeneration by affecting caspase-dependent apoptosis [12]. The ECR is activated by 20-hydroxyecdysone (20E), a commonly occurring steroid hormone, and coordinates multiple developmental events, including molting, diapause, spermatogenesis, and salivary gland degeneration. In addition, studies of ticks have shown that the let-7 microRNA can regulate the expression of the ECR to influence molting [13]. However, the mechanism by which the ECR coordinates salivary gland degeneration remains unknown.

MicroRNAs (miRNAs) are short regulatory noncoding small RNAs that regulate the expression of target genes at transcriptional and posttranscriptional levels [14, 15]. miRNAs are involved in various biological processes in ticks and other arthropods, including blood feeding, spawning, molting, and development [16–20]. In a study of *Drosophila*, mir-34, mir-100, mir-125, and let-7 underwent temporal changes at different developmental stages and responded to 20E or JH (juvenile hormone) in S2 cells [21]. Furthermore, it was found that iri-miR-34-5p is highly expressed in salivary glands in the *Ixodes ricinus* saliva as analyzed by silico target network analysis of de novo-discovered [22]. In our study, deep sequencing of *R. haemaphysaloides* identified several miRNAs expressed at different developmental stages of the salivary glands. Among these miRNAs, miR-34-5p was highly expressed at the feeding blood meal stages, and it is highly homologous to iri-miR-34-5p. 

On the basis of this finding, we hypothesized that miR-34-5p may play a crucial role in the development of *R. haemaphysaloides*. Therefore, the present study investigated the biological role of miR-34-5p in the degeneration of the salivary glands of *R. haemaphysaloides*.

## Methods

### Tick feeding and tissue collection

Adult *R. haemaphysaloides* were collected in May 2001 from a water baffle in Wuhan Hubei Province, China. The ticks fed on the ears of New Zealand white rabbits (SLAC, Shanghai Institutes for Biological Science, CAS) and were maintained in artificial climate incubators at the Shanghai Veterinary Research Institute [23]. The salivary gland tissues were rapidly dissected, washed with phosphate-buffered saline (PBS; PH 7.4; with 0.14 M NaCl, 0.0027 M KCl, and 0.01 M phosphate buffer; Gibco, Life Technologies, Carlsbad, CA, USA), and placed in PBS or TRIzol (Invitrogen, California, USA) reagent at −80 °C. 

### RNA extraction and cDNA synthesis

RNA was derived from salivary glands of ticks dissected at different feeding stages (unfed and fed for 5 days and engorged for 3 days) and preserved in TRIzol reagent. To synthesize cDNAs, 1 µg of total RNA was reverse transcribed using a Prime Script™ RT reagent kit with gDNA Eraser (TaKaRa, Shiga, Japan), using stem-loop primers for miR-34-5p or oligo dT (18) for the RhECR and Rhcaspase7 gene.

### Small RNA deep sequencing

Salivary glands of both unfed ticks, ticks that fed for 5 days, and ticks that were engorged for 3 days were used for total RNA extraction. RNA purification, reverse transcription, library construction, and sequencing were performed at Shanghai Gene Denovo Biotechnology Co. (Guangzhou, China) according to the manufacturer’s instructions. A total amount of 1 μg total RNA per group sample was used as input material for the small RNA library. Sequencing libraries were generated using QIAseq miRNA Library Kit (Qiagen, Germany) following manufacturer’s recommendations. The activated 5′ and 3′ adaptors were ligated to the total RNA, respectively. Then the adaptor-ligated RNA was transcribed into first-strand cDNA by using reverse transcriptase and random primer. A polymerase chain reaction (PCR) reaction was performed using primers complementary for 11–12 cycles, and fragments of appropriate size were isolated by a 6% Novex TBE PAGE gel. After being quantified by Qubit 4.0, the sequencing library was performed on NovaSeq X Plus platform using NovaSeq Reagent Kit. The reference genome for comparison is *Rhipicephalus sanguineus* (ncbi_GCF_013339695.2).

### Prediction of target sites

Target gene predictions for miR-34-5p were performed with miRanda [24] for animal and RNAhybrid [25, 26]. The predicted target genes, from the salivary glands transcriptome of *R. haemaphysaloides* [11], were annotated with Gene Ontology (GO) (http://www.geneontology.org/) and Kyoto Encyclopedia of Genes and Genomes (KEGG) (http://www.genome.jp/kegg/) databases. Functional-enrichment analysis including GO and KEGG were performed to identify which targets were significantly enriched in GO terms and metabolic pathways at *P*-adjust < 0.05 compared with the whole-ref genes background. GO functional enrichment and KEGG pathway analysis were carried out by Goatools (version 0.6.5) and Python scipy (version 1.15.0) software, respectively.

### Amplification of the 3′‑UTR region of RhECR

Total RNA was applied for cloning 3′ cDNA end sequences using a HiScript-TS 5′/3′ RACE Kit (Vazyme Biotech, Nanjing, China) according to the protocol provided in the kit with specific primers (Additional File 1: Supplementary Table S2). The 3′‑UTR region of RhECR was purified and cloned into the PMD 19-T vector (Takara, Shiga, Japan). The amplified fragment was identified by Sanger sequencing.

### Dual luciferase reporter assay

An miR-34-5p mimic and a negative control (NC) were synthesized by Sangon (Shanghai, China). The miRNA mimics are chemically modified double-stranded RNA, and mimic endogenous miRNAs were used to verify the relationship between the target genes and miRNAs. The miRNA negative control was a mimic dissimilar to mammalian and tick miRNAs. The 487-bp RhECR wild-type (WT) or mutant 3′‑UTR were separately cloned into the *Xba*I and *Pme*I sites of a pmirGLO vector (Promega, Madison, USA).

A mammalian HEK293T cell line was used for the DLR assay owing to its high transfection efficiency and low background expression of target genes. HEK293T cells were maintained in Dulbecco’s Modified Eagle Medium (DMEM, Gibco, Life Technologies, Carlsbad, CA, USA), supplemented with 10% heat-inactivated fetal bovine serum (Biological Industries, Kibbutz Beit Haemek, Israel) and 1% penicillin (Gibco, Life Technologies, Carlsbad, CA, USA) at 37 °C. The HEK293T cells were co-transfected with 0.2 ng of the pmirGLO-target reporter vector and 50 nM miRNA mimic and mixed with 2 μL of Lipofectamine 3000 transfection reagent (Invitrogen, California, USA) and 50 μL of Opti-MEM Reduced Serum Medium (Gibco, Life Technologies, Carlsbad, CA, USA) in each well of a 24-well plate.

The activities of the firefly and renilla luciferases were measured using a Dual-Luciferase Reporter Assay System (Vazyme Biotech, Nanjing, China). Results are shown as the ratio of Renilla/firefly luciferase activity (mean ± standard error (SEM)). Each sample was performed in triplicate, and transfections were repeated three times.

### Real‑time quantitative PCR (RT-qPCR)

The expression levels of miR-34-5p, RhECR and Rhcaspase7 were estimated by RT-qPCR conducted using ChamQ Universal SYBR qPCR Master Mix (Vazyme Biotech, Nanjing, China) green and gene-specific primers (Additional File 1: Supplementary Table S1) with a QuantStudio 5 PCR System (Applied Biosystems, Austin, TX, USA). The RT-qPCR process consisted of 95 °C for 30 s, followed by 40 cycles at 95 °C for 5 s and 60 °C for 30 s and a final analysis of the melting curve. All samples were analyzed in triplicate. The data used elongation factor-1(ELF1A, GenBank accession no. AB836665) as an internal control, and this was used to analyze the relative gene expression in each sample using the 2^−ΔΔCt^ method.

For the analysis of the expression of miR-34-5p and RhECR, the salivary gland tissues were rapidly dissected in normal female ticks at different feeding stages (unfed, fed for 3 and 5 days, and engorged for 0 and 3 days) and preserved in TRIzol reagent.

### Application of AgomiR and AntagomiR mimic

To further validate the role of miR-34-5p, miRNA was overexpressed in vivo using the miR-34-5p AgomiR and AntagomiR. An AgomiR is a chemically modified double-strand miRNA mimic. AntagomiRs, also known as anti-miRNAs or blockmirs, are a class of chemically engineered oligonucleotides that prevent other molecules from binding to a desired site on an mRNA molecule. AntagomiRs are used to silence endogenous microRNAs [27].

We interfered with the miRNA in the salivary glands in vitro using the AgomiR-34-5p or AntagomiR-34-5p [28]. The control group was treated with AgomiR-NC or AntAgomiR-NC. The salivary glands of female ticks fed on rabbits for 5 days were dissected and placed into a 24-well plate containing complete L15 medium with 1% penicillin–streptomycin [29]. Each well received 5 μM of the mimic (AgomiR-34-5p, AntagomiR-34-5p, AgomiR-NC, or AntAgomiR-NC). The plates were incubated at 27 °C without CO_2_ for 48 h. Each treatment group included the salivary glands from ten ticks. After incubation, three pairs of the salivary glands in each group were fixed in 4% paraformaldehyde for hematoxylin and eosin (H&E) and Terminal deoxynucleotidyl transferase dUTP Nick-End Labeling (TUNEL) staining for at least 24 h at 4 °C, which verifies the degree of apoptosis in salivary gland. The remaining salivary glands were used for RNA extraction for RT-qPCR analysis, which verifies the expression of RhECR and RhCaspase7. 

To verify the effect of miR-34-5p in vivo, unfed female ticks (*n* = 30 females per group) were microinjected with approximately 100 nmol of AgomiR-34-5p [20]. Control ticks were injected with 100 nmol of AgomiR-NC. After mimic injection, the ticks were kept in a dark chamber at 27 ℃ and 95% humidity for 1 day, after which they were allowed to feed on rabbit ears until they were fully engorged. Each group of ticks was allowed to feed on three rabbits (30 ticks on each rabbit). RT-qPCR was used to evaluate the efficiency of RhECR gene silencing. The biological parameters analyzed were the feeding period (the number of days the females were attached to rabbit ears) and the engorgement weight (the weight of the tick when it falls off the rabbit’s ear).

### RNA interference and rescue experiments

RNAi experiments were designed against RhECR genes. The RhECR sequences were screened using Primer Premier 5 for designing RNAi primers. Specific primers (Additional File 1: Supplementary Table S4) containing the T7 polymerase promoter sequence were used for PCR amplification. The amplicons were then purified to obtain templates for double-stranded RNA synthesis using the T7 RiboMAX Express RNAi system (Promega, Madison, WI, USA). The unrelated Luciferase dsRNA was synthesized using the same methods described previously and used as the negative control.

In the rescue experiments, the unfed female ticks (*n* = 30 females per group) were divided into four groups and treated as follows: co-injection with 0.5 μL of an AntagomiR34-5p and dsRhECR mixture (100 nmol AntagomiR34-5p and 1μg dsRhECR); co-injection 0.5 μL of an AntAgomiR-NC and dsLuciferase mixture (100 nmol AntAgomiR-NC and 1 μg dsLuciferase); injection with 1 μg dsRhECR; and injection with 1 μg dsLuciferase.

After injection, the ticks were kept in a dark chamber at 27 ℃ and 95% humidity for 1 day, after which they were allowed to feed on rabbit ears until they were fully engorged. The salivary gland tissues were rapidly dissected on the 5th day of feeding blood. Three pairs of the salivary glands in each group were fixed in 4% paraformaldehyde for H&E and TUNEL staining for at least 24 h at 4 °C, which verifies the degree of apoptosis in salivary gland. The remaining salivary glands were used for RNA extraction for RT-qPCR analysis, which verifies the expression of RhECR.

### TUNEL staining and H&E staining

The salivary glands of female ticks were fixed in 4% formalin and paraffin-embedded. Sections of the salivary glands were mounted on microscope slides. The tissue sections were then deparaffinized, washed in 100% ethanol, and rehydrated. The samples were washed with PBS. After antigen retrieval with 0.1% Triton X-100, the tissues were incubated for 1 h with 1:9 TdT mixed with fluorescent-labeled dUTP at 37 °C, following the instructions of the Roche in Situ Cell Death Detection Kit, POD (Roche, Mannheim, Germany). After washing the sections two or three times with PBS, the sections were stained with 1 μg/mL 4′, 6′-diamidino-2-phenylindole (DAPI, Invitrogen, California, USA) in distilled H_2_O for 20 min [11]. After washing, the sections were mounted using Lab Vision™ PermaFluor™ (Invitrogen, California, USA) medium under glass coverslips, then viewed and photographed on a Pannoramic DESK Digital Slide Scanner (3D HISTECH, Budapest, Hungary) [11].

Salivary glands were fixed in 4% paraformaldehyde and embedded with paraffin. Paraffin sections were stained with hematoxylin and eosin (H&E). H&E staining images were obtained by light microscopy (Nikon Eclipse 80i microscopy system using a 40X objective).

### Colocalization of miR-34-5p and RhECR by fluorescence in situ hybridization

For the assessment of miR-34-5p and RhECR colocalization, antisense RNA detection probes targeting miR-34-5p and RhECR gene were designed and labeled with the dual fluorophores Cy3 and FAM, respectively, at Servicebio Biotechnology Company (Wuhan, China). A scrambled sequence and the sense probe for the target gene were used as NC groups. Then, 5 days after feeding on blood, the tick salivary glands were dissected in cold PBS buffer, fixed overnight in 4% paraformaldehyde, and incubated overnight at 37 °C for probe hybridization. The tick samples were washed in PBS containing 5% Triton X-100 (*v*/*v*) for 10 min and stained with DAPI G1012 (Servicebio, Wuhan, China) at room temperature for 8 min. The signals of miR-34-5p and RhECR were detected using an Eclipse CI upright fluorescence microscope (Nikon, Japan). The probes for the miRNA and target gene are listed in Additional File 1: Supplementary Table S5.

### Data analysis

All statistical analyses were performed using GraphPad Prism 6.0 software (Graph Pad Software Inc., San Diego, CA, USA). Mean ± standard error (SEM) values were calculated for three separate experiments, and two-tailed Student’s *t*-tests were used to identify significant differences between groups. *P* < 0.05 was considered statistically significant.

## Results

### Small RNA analysis of tick salivary glands

**Fig. 1:**
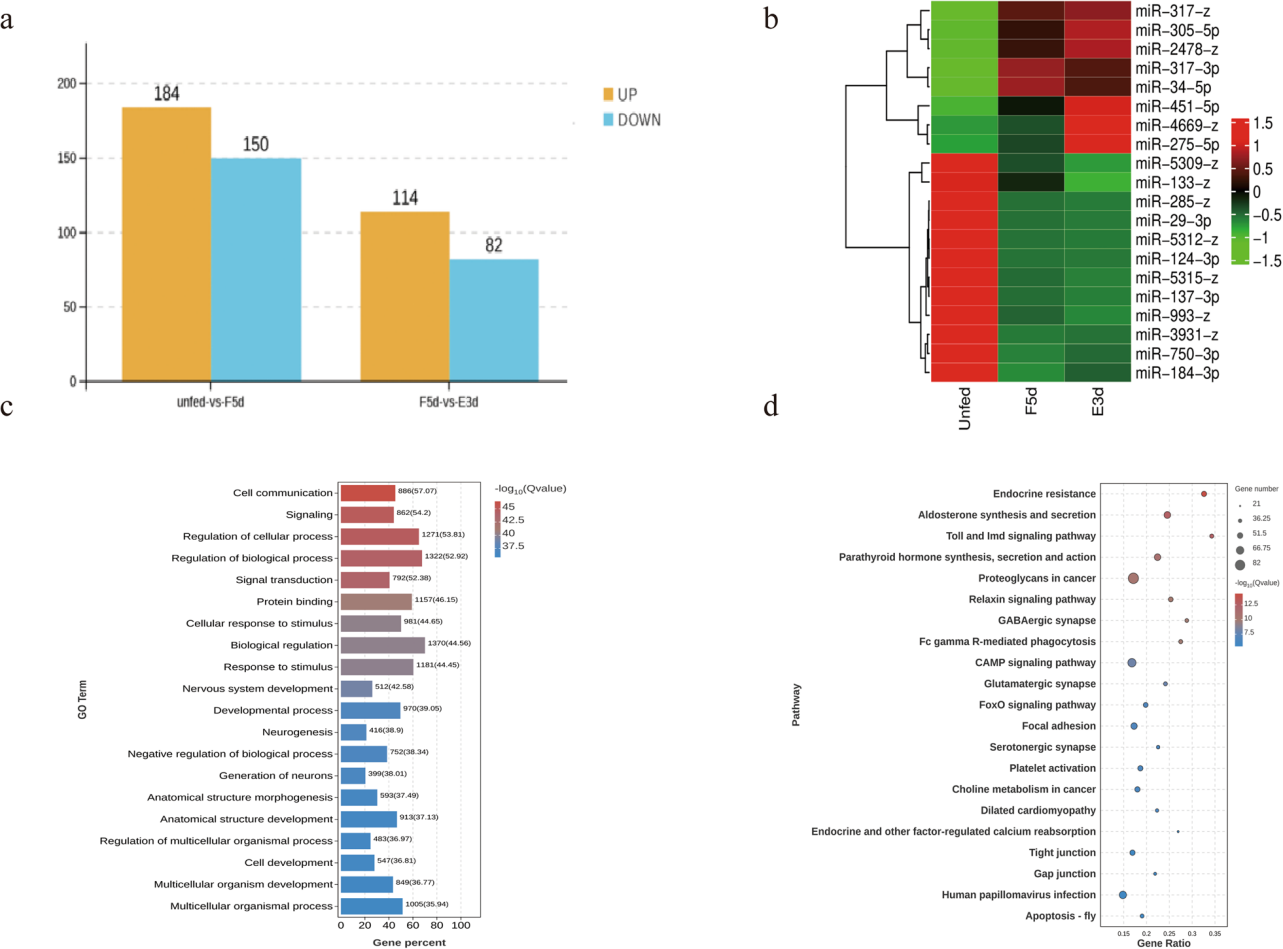
Small RNA analysis of tick salivary glands. **a** Differential gene analysis of unfed, F5d, and E3d groups. **b** Cluster heatmap of unfed, F5d, and E3d groups. F5d: fed for 5 days; E3d: engorged for 3 days. **c** GO functional enrichment of targets from miR-34-5p. **d** KEGG functional enrichment of targets from miR-34-5p

We compared and analyzed the salivary glands of female ticks at different blood-sucking stages to study the potential functions of miRNAs. The period from before the initiation of blood feeding (unfed) to day 5 of feeding (F5d) involves the rapid development of the salivary glands, and from day 5 of feeding to day 3 of engorgement (E3d) the salivary glands rapidly degenerate. In the comparison between unfed ticks and the F5d group, 184 genes were significantly upregulated, and 150 genes were significantly downregulated. In the comparison between the F5d group and the E3d group, 114 genes were significantly upregulated, and 80 genes were significantly downregulated (Fig. 1a). A heatmap analysis was employed to screen the 20 miRNAs with the greatest difference in expression, and miR-34-5p had the most significant difference (Fig. 1b). GO enrichment analysis of the predicted targets of miR-34-5p shows an important biological process pathway (Fig. 1c). Among these pathways, we were intrigued by the biological regulation and development process, with 1370 and 970 genes, respectively (Additional File 2). KEGG enrichment analysis showed that functional signaling pathways were enriched by these target genes (Fig. 1d). We found that apoptosis genes were significantly expressed in these target genes of miR-34-5p. The predicted targets of miR-34-5p are listed in Additional File 2.

### miR-34-5p targets RhECR in vitro

**Fig. 2:**
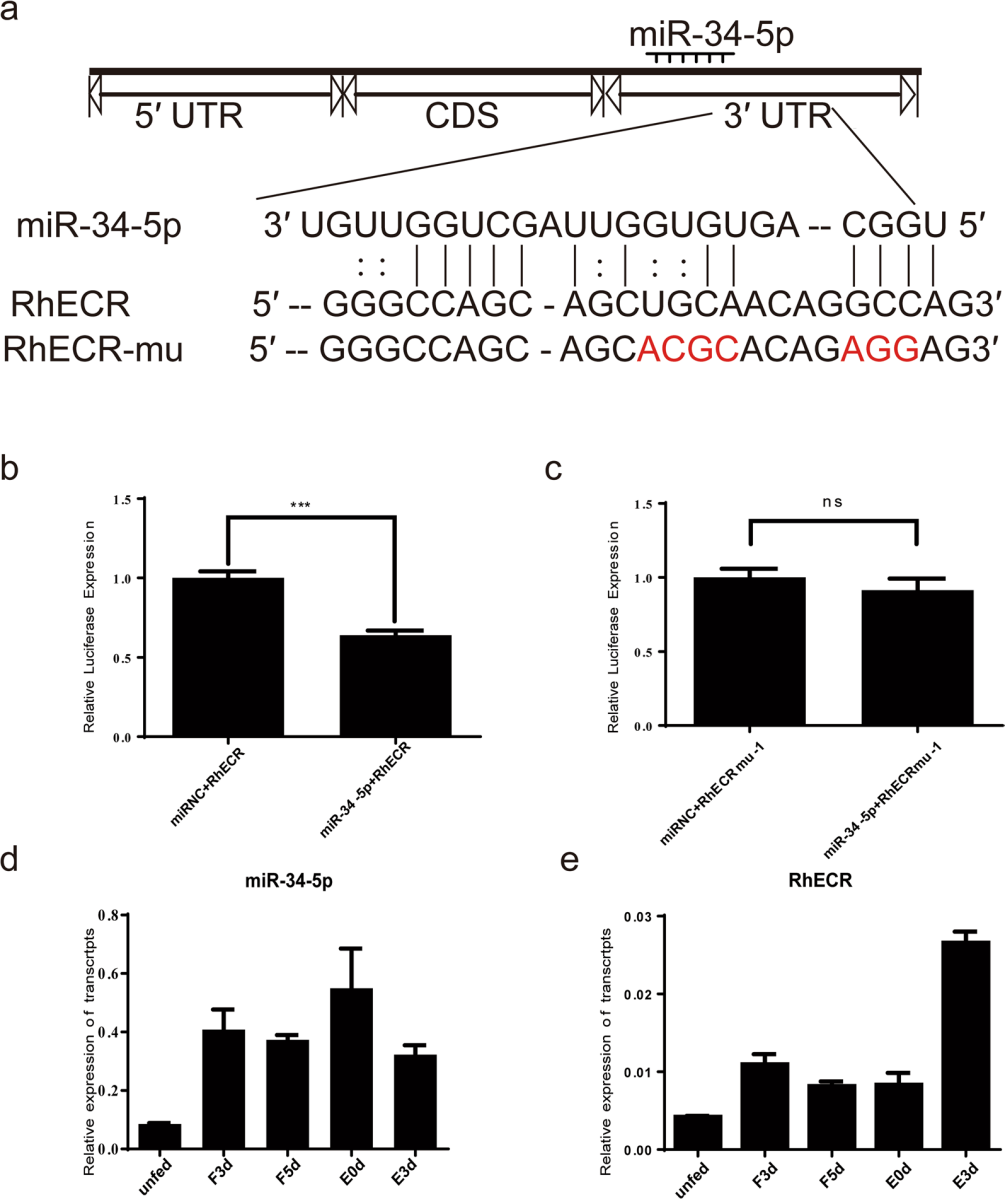
Target gene prediction of miR-34-5p. **a** Sequence alignments of miR-34-5p with the target gene RhECR and the miRNA-resistant mutated version at the predicted binding sites (RhECR-mu). Mutated nucleotides are shown in red. **b**, **c** RhECR is a target of miR-34-5p. Dual luciferase reporter assay results are representative of triplicate samples. Values are expressed as mean ± SEM. **P < 0.01 and ***P < 0.001; ****P < 0.0001; *ns* not significant; (Student’s *t*-test). **d**, **e** RT-qPCR analysis of miR-34-5p and RhECR gene expression during different feeding stages

We used three stringent miRNA target prediction software programs (miRanda and RNAhybrid) to predict potential targets of miR-34-5p in the 3′ UTR. The analysis identified a potential binding site in RhECR (Fig. 2a). On the basis of the results, the binding sites were cloned and inserted downstream of Renilla in the pmirGLO vector, which was then cotransfected into 293 T cells with miR-34-5p mimics. To further examine whether RhECR gene expression could be specifically affected by miR-34-5p, we generated an RhECR mutation (RhECR-mu) at the miRNA binding sites. Dual luciferase reporter assays revealed that miR-34-5p significantly silenced the target gene RhECR (Fig. 2b) but did not suppress the RhECR mutant compared with the NC groups (Fig. 2c). These results suggest that RhECR genes were targeted by the miR-34-5p.

### Analysis of the expression of miR-34-5p and RhECR

The cDNA of the salivary glands of adult female ticks at the feeding stage was subjected to RT-qPCR to evaluate the expression profiles of miR-34-5p and RhECR genes during the feeding stages. During salivary gland degeneration (E0d–E3d), the expression level of miR-34-5p decreased (Fig. 2d), but RhECR maintained a high expression level (Fig. 2e). Therefore, the analysis suggested that there is an interaction between miR-34-5p and ECR and that this interaction affects salivary gland degeneration.

### Colocalization of the miR-34-5p and RhECR

**Fig. 3:**
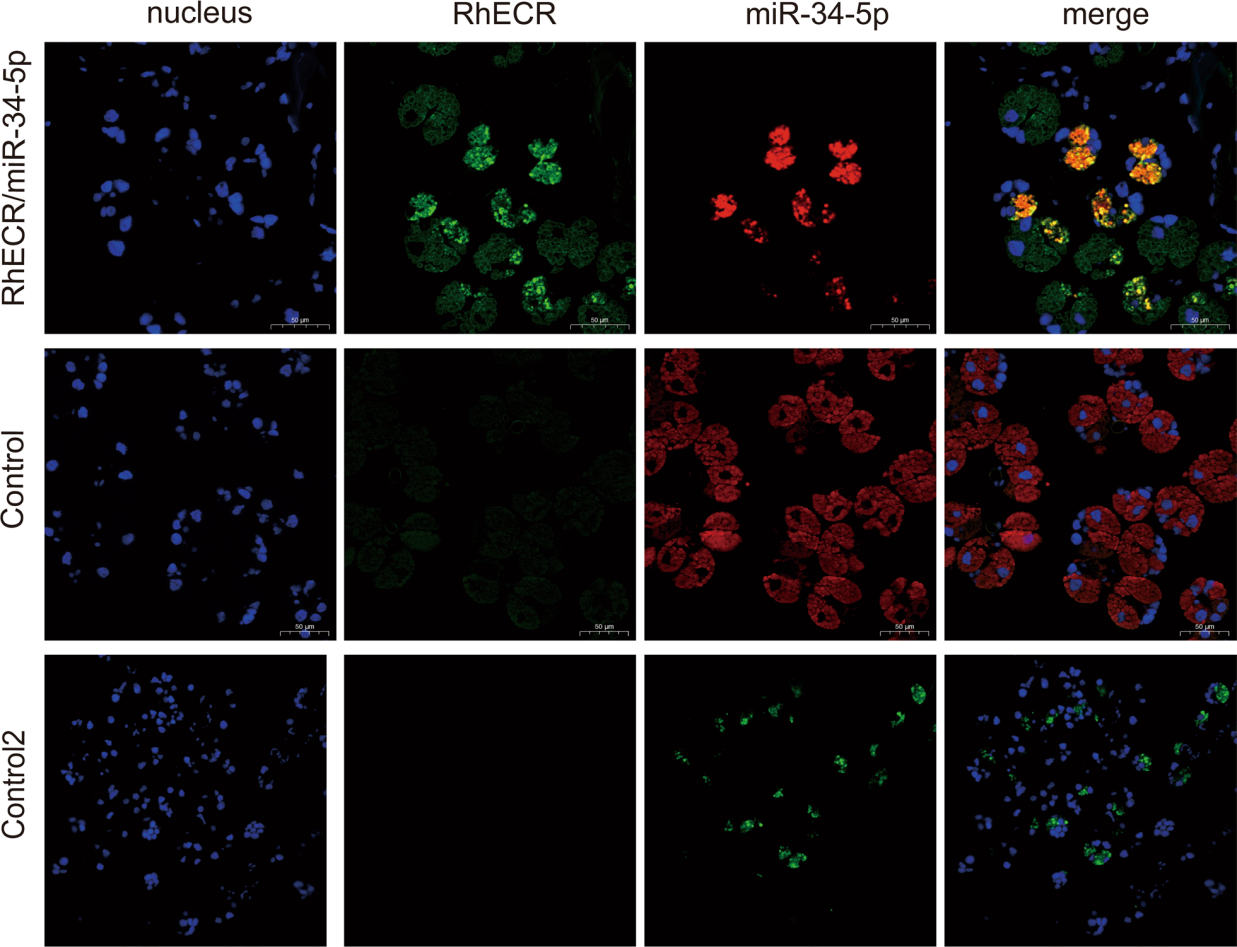
Colocalization of miR-34-5p with the target gene RhECR in the salivary glands of ticks. Localization of miR-34-5p and RhECR in the salivary gland cells was detected by fluorescence in situ hybridization (FISH). Nuclei were stained with DAPI; miR-34-5p probes were labeled with Cy3 (red); and RhECR probes were labeled with FAM (green)

To determine whether miR-34-5p and RhECR were colocalized in the salivary glands of female ticks, we performed in situ analyses of miR-34-5p and RhECR via miRNA/mRNA fluorescence in situ hybridization. Both miR-34-5p and RhECR were detected in the salivary glands of the ticks (Fig. 3), suggesting that RhECR interacts directly with miR-34-5p in the tick salivary gland in a spatially dependent manner.

### MiR-34-5p mimic results in the degeneration of tick salivary glands in vitro

**Fig. 4:**
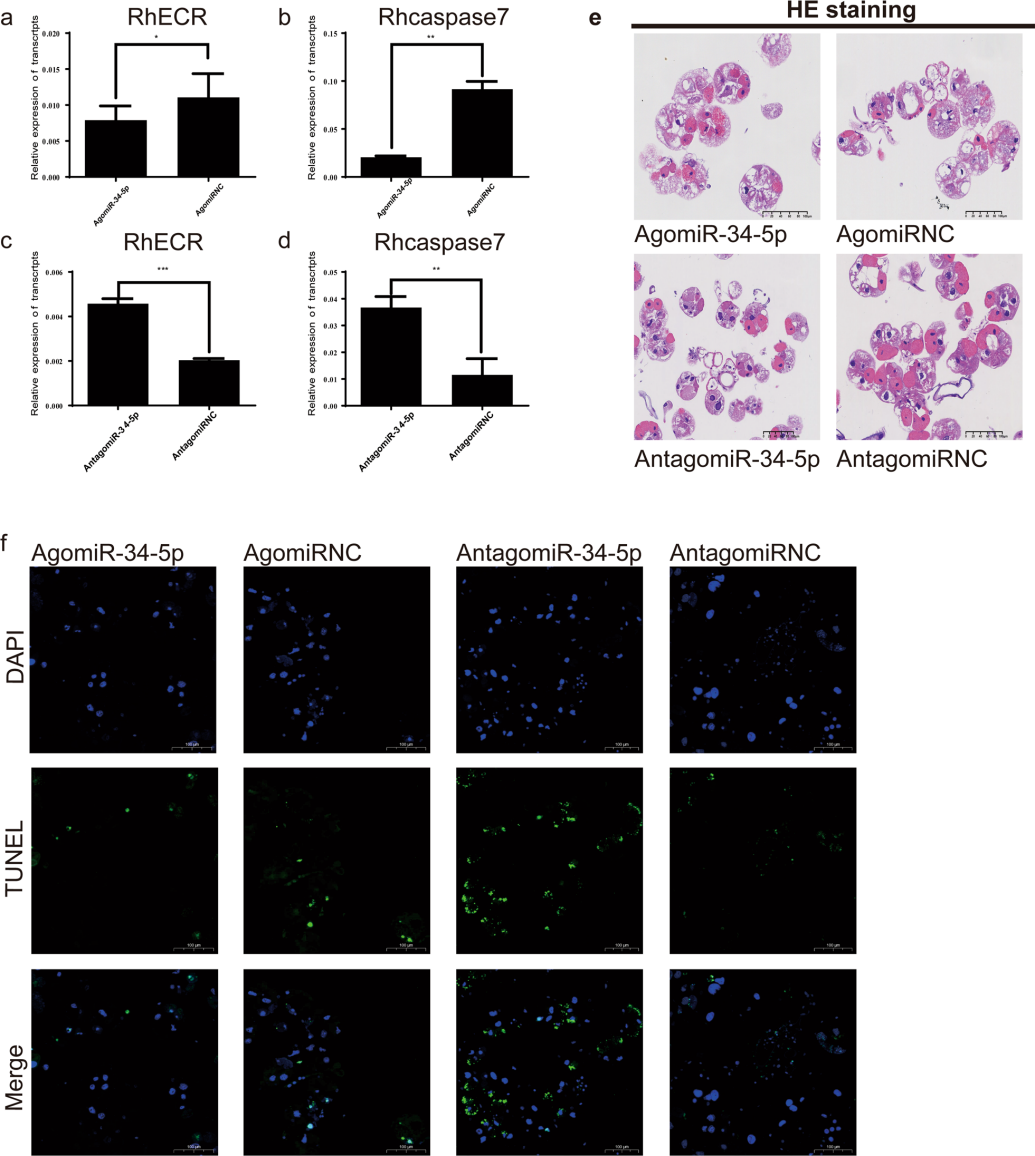
The effect of miR-34-5p in vitro interference on tick salivary gland degeneration. **a**, **b** RhECR and Rhcaspase7 expression levels in the salivary glands were determined after interference with AgomiR-34-5p and AntagomiR-34-5p. **c**, **d** RhECR and Rhcaspase7 expression levels in the salivary glands were determined after interference with AntagomiR-34-5p. **e** H&E staining assays of apoptosis levels after interference by AgomiR-34-5p and AntagomiR-34-5p. **f** TUNEL staining assays of apoptosis levels after interference by AgomiR-34-5p and AntagomiR-34-5p. **P < 0.01; ***P < 0.001; ****P < 0.0001; *ns* not significant (Student’s *t*-test)

To verify the role of miR-34-5p in the degeneration of the salivary glands, we altered the expression of miR-34-5p by using an AgomiR or AntagomiR in salivary glands of *R. haemaphysaloides* female adults and assessed the transcript levels of RhECR after incubation at 27 °C without CO_2_ for 48 h in vitro. The qRT-PCR results showed that the mRNA expression levels of RhECR and RhCaspase7 were decreased in the AgomiR-treated group compared with the AgomiR-NC group (Fig. 4a, b). However, RhECR and RhCaspase7 were increased in the AntagomiR-treated group compared with the AgomiR-NC groups (Fig. 4c, d), suggesting that the stimulation of miR-34-5p expression could downregulate RhECR expression to inhibit apoptosis. The salivary glands of AgomiR-treated ticks showed unbroken acini compared with the AgomiR-NC ticks by H&E staining (Fig. 4e). TUNEL assays showed that salivary gland interference by AgomiR treatment decreased apoptosis levels compared with the AgomiR-NC group; however, the AntagomiR-treated group demonstrated the opposite result (Fig. 4f).

### Additional miR-34-5p affects tick feeding behavior

**Fig. 5:**
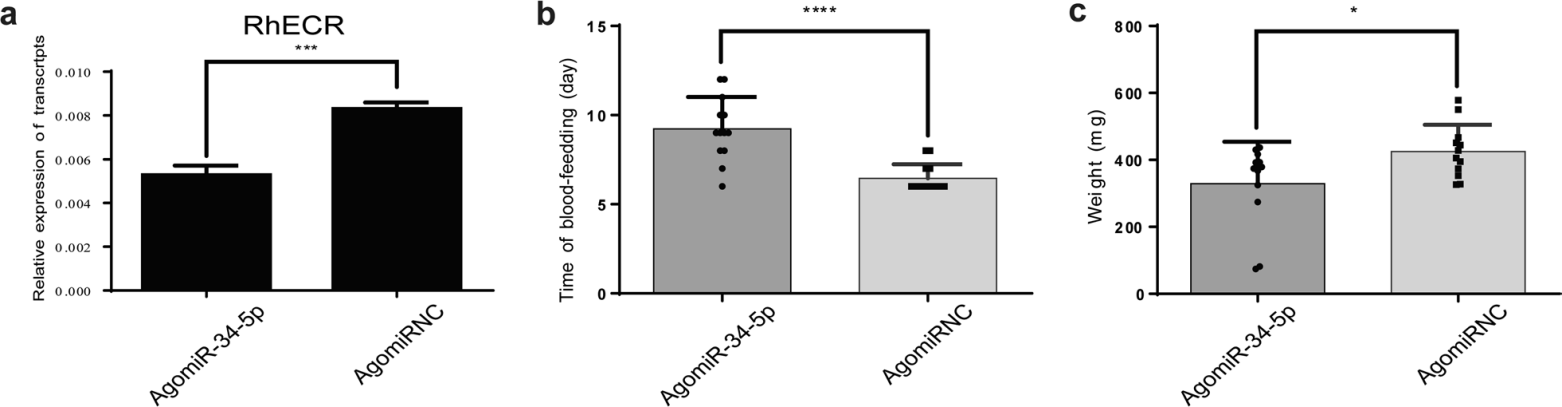
The overexpression of miR-34-5p significantly suppressed the expression of RhECR and delayed the feeding period in vivo. **a** RhECR expression levels in the salivary glands were determined after injecting AgomiR-34-5p. **b** Time of satiation in adult females after miR-34-5p overexpression. **c** Weight of saturated adult ticks following miR-34-5p overexpression. **P < 0.01 and ***P < 0.001; ****P < 0.0001; *ns* not significant (Student’s *t*-test)

To identify the function of miR-34-5p, we microinjected an AgomiR into adult female *R. haemaphysaloides* before they were fed a blood meal. The transcript levels of RhECR were assessed at 3 days after the engorgement. The qRT-PCR results showed that the mRNA level of RhECR was significantly reduced in the AgomiR-treated group compared with the AgomiR-NC group (Fig. 5a). However, the blood meal feeding period was extended by 3 days in the AgomiR-treated group (Fig. 5b), and the weight of AgomiR-treatment was decreased compared with the AgomiR-NC group (Fig. 5c).

### Phenotype rescue confirms that RhECR is a direct target of miR-34-5p in vivo

**Fig. 6:**
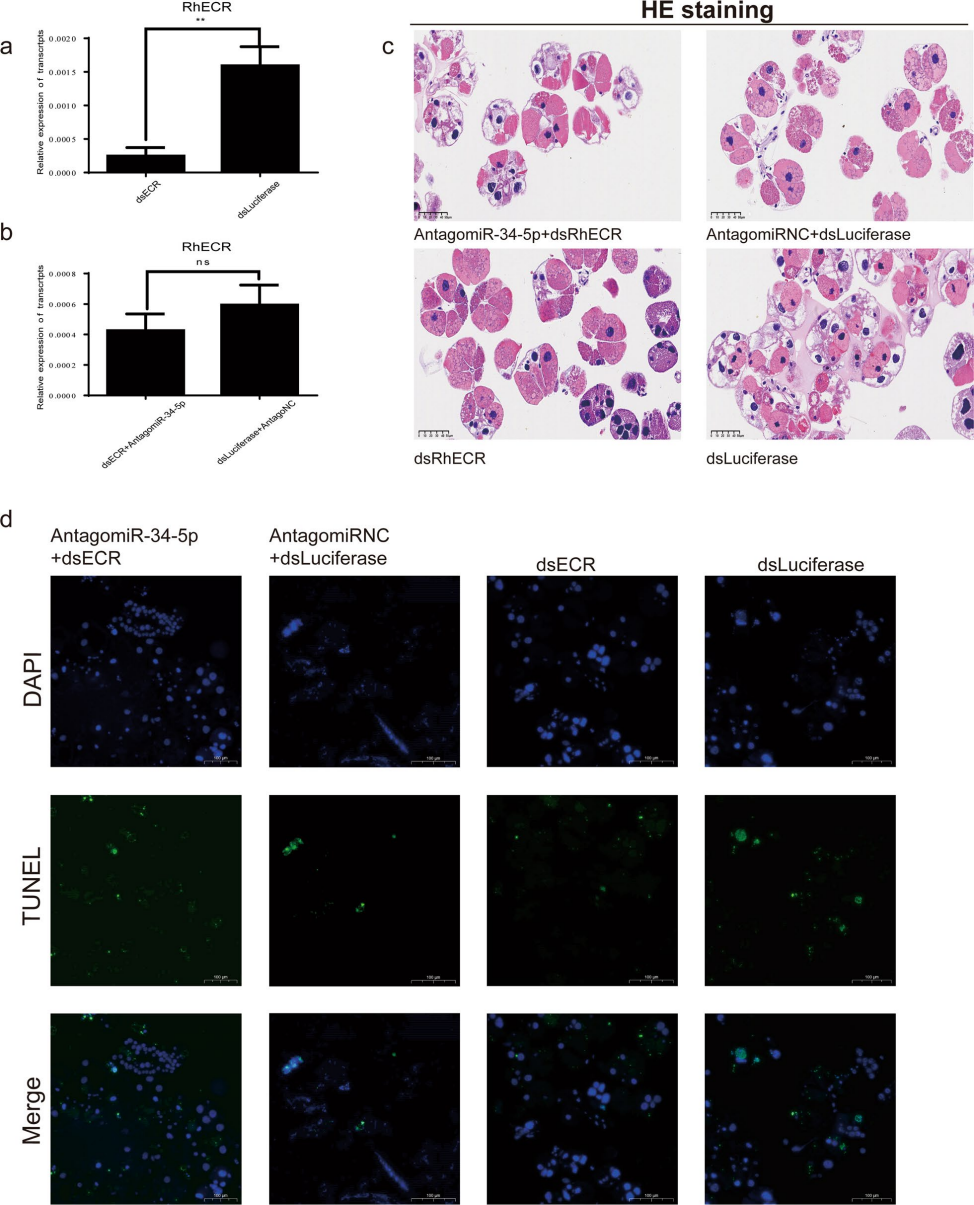
miR-34-5p partially rescued the RhECR-dsRNA phenotypes. **a**, **b** RhECR expression levels in the salivary glands were determined after injecting AntagomiR-34-5p and dsRNA-RhECR. **c** H&E staining assays for apoptosis levels after injecting the salivary glands with AntagomiR-34-5p and dsRNA-RhECR. **d** TUNEL staining assays of apoptosis levels after injecting the salivary glands with AntagomiR-34-5p and dsRNA-RhECR. **P < 0.01 and ***P < 0.001; ****P < 0.0001; *ns* not significant (Student’s *t*-test)

To verify whether RhECR was the target of miR-34-5p during the degeneration of *R. haemaphysaloides* salivary glands, a behavioral phenotype rescue experiment was performed by microinjecting an AntagomiR to block miR-34-5p expression and using designed dsRNA for the RNAi-mediated depletion of the RhECR gene in *R. haemaphysaloides* before being fed a blood meal. The qRT-PCR results showed that the RhECR mRNA level was significantly reduced in the dsRNA-treated group compared with the dsLuciferase control groups (Fig. 6a), but the AntagomiR34 and dsRhECR groups had no significant difference compared with the AntAgomiR-NC and dsLuciferase groups (Fig. 6b). H&E staining revealed that the AntagomiR34 and dsRhECR-treated groups had more broken salivary gland acini than the dsRhECR-treated group, but the salivary glands were more complete compared with the AntAgomiR-NC and dsLuciferase or only dsLuciferase groups (Fig. 6c). TUNEL assays showed that the AntagomiR34 and dsRhECR-treated groups had more apoptotic fragmentation than the dsRhECR-treated group, but lower apoptosis levels compared with AntAgomiR-NC and dsLuciferase or only dsLuciferase treatments (Fig. 6d). The results suggested that the RNAi-mediated silencing of RhECR resulted in complete gland acini resulting from the suppression of salivary gland degeneration; however, apoptosis was restored by silencing miR-34-5p. Hence, the phenotypic rescue achieved by the administration of RhECR RNAi to miR-34-5p-depleted *R. haemaphysaloides* salivary glands confirmed that RhECR was an authentic target of miR-34-5p in vivo*.*

## Discussion

*Rhipicephalus haemaphysaloides* is a three-host hard tick widely distributed in China and an important vector of infectious pathogens [30–32]. Tick-borne pathogens (TBPs) are transmitted to hosts through tick bites assisted by saliva [3, 4]. The salivary glands of female ticks undergo degeneration after engorgement [5]. In our previous research, we found that apoptosis plays an important role in this process, with significant changes in the expression levels of caspase genes and apoptosis-related genes (RhIAP, RhBcl-2, and RhBax) [11, 28, 33]. In addition, the ECR promotes salivary gland degeneration by affecting caspase-dependent apoptosis [12]. In a study of *Drosophila*, 20E-induced broad-complex could regulate the miRNAs (let-7, miR-100, miR-34, and miR-125) involved in the 20E signaling pathway [21], but the mechanism was unknown. In this study, we identified and characterized miR-34-5p and its target gene RhECR in *R. haemaphysaloides* and found that they were involved in salivary gland degeneration.

Salivary gland degeneration is triggered after the completion of female tick feeding. The RT-qPCR results showed that during salivary gland degeneration, the expression level of miR-34-5p remained at low levels, but RhECR maintained at a high expression level. These results suggested that miR-34-5p and RhECR were inversely related expression levels during salivary gland degeneration in *R. haemaphysaloides*.

We used miRNA target prediction software to predict potential targets of miR-34-5p. The results identified a potential binding site in the RhECR 3′UTR. Co-transfection of a luciferase gene vector containing RhECR 3′UTR with miR-34-5p mimics resulted in decreased luciferase activity in vitro as determined by a dual luciferase reporter assay, suggesting that RhECR is a direct target gene of miR-34-5p. Furthermore, the miRNA/mRNA fluorescence in situ hybridization results showed that in the tick salivary gland, RhECR directly interacted with miR-34-5p in a spatially dependent manner.

RNAi was used to study the physiological roles of RhECR. H&E and TUNEL staining were used to evaluate the status of salivary gland acini and the rate of DNA fragmentation in degenerated tick salivary glands. H&E staining showed that compared with the control group, integrity of the acini of the salivary glands was higher, and the positive staining rate in the TUNEL assay was decreased significantly by interfering with dsRNA-RhECR. These results suggested that RhECR functions in accelerating apoptosis during the degeneration of the salivary glands. The overexpression of miR-34-5p decreased RhECR expression and significantly delayed the blood meal feeding period of *R. haemaphysaloides*. Meanwhile, the acini cell apoptosis in the salivary glands was inhibited compared with the control group. However，AntagomiR-mediated inhibition of miR-34-5p upregulated RhECR expression, thereby promoting apoptosis. 

The phenotypic rescue experiment showed that in the antagomimic and dsRhECR-treated groups, apoptosis of the salivary gland acini cells was accelerated compared with the dsRhECR-treated group, but was decreased compared with the control group. These results indicated that the AntagomiR could bind to miR-34-5p in vivo and counteract the inhibition of miR-34-5p; thus, only the inhibitory effect of the dsRhECR was reflected in the phenotype. In sum, the results indicate that RhECR promotes salivary gland degeneration, while miR-34-5p has a significant inhibitory effect on RhECR, suggesting that RhECR is a target gene of miR-34-5p in vivo.

Previous studies have shown that ECR play important physiological regulatory roles not only in ticks but also in other arthropods, such as mosquitoes, silkworms, and drosophilas [34–37]. We demonstrated the critical regulatory role of ECR in tick feeding processes [12]. Now, we have discovered that miRNAs are also involved in regulating tick blood-feeding behavior. Specifically, we found that miRN-34-5p influences salivary gland development by modulating RhECR expression in *R. haemaphysaloides*, thereby further affecting the tick’s blood-feeding efficiency. Owing to their ease of chemical synthesis and resistance to endogenous nucleases, miRNAs offer significant potential for manipulating ncRNA (non-coding RNA)-mediated regulation of insect behavior, providing a promising avenue for novel pest control strategies [19]. For instance, miRNA-based approaches, such as short tandem target mimic (STTM) technology expressed in transgenic plants, have been successfully used to suppress locust swarm formation [38].

## Conclusions

Our study describes a novel mechanism by which miR-34-5p regulates the expression of RhECR during the degeneration of the salivary glands in *R. haemaphysaloides* ticks. During the blood-feeding period, the inhibition of RhECR expression by miR-34-5p promotes the rapid development of the salivary glands. However, during tick engorgement, the expression level of miR-34-5p decreases; RhECR is highly expressed, promoting the rapid degeneration of the salivary glands. This study provides new perspectives concerning the involvement of miRNA in the degeneration of tick salivary glands through its effect on target genes. The findings suggest new ideas for the prevention and control of ticks and tick-borne diseases.

## Supplementary Information

Additional File 1: Supplementary Table S1. Primers used for quantitative real-time polymerase chain reactions of* R. haemaphysaloides* RhECR genes and miR-34-5p. Table S2. Primers for* R. haemaphysaloides* RhECR 3′UTR region and Dual luciferase vector cloning. Table S3. Primers used for overlap extension (SOE) of recombinant PCR to mutant binding sites. Table S4. Primers for RNAi of* R. haemaphysaloides* RhECR genes. Table S5. The probes for the miRNA and target gene about in situ hybridization.

Additional File 2: The identified miRNAs with their expression levels and GO or KEGG analysis of the predicted targets of miR-34-5p.

## Data Availability

Data are provided within the manuscript or supplementary information files.

## References

[1] Jongejan F, Uilenberg G. The global importance of ticks. Parasitology. 2004;129:S3-14. 15938502 10.1017/s0031182004005967

[2] Sonenshine DE. Biology of ticks. New York: Oxford University Press; 1991.

[3] Simo L, Kazimirova M, Richardson J, Bonnet SI. The essential role of tick salivary glands and saliva in tick feeding and pathogen transmission. Front Cell Infect Microbiol. 2017;7:281. 28690983 10.3389/fcimb.2017.00281 PMC5479950

[4] Kazimírová M, Stibrániová I. Tick salivary compounds: their role in modulation of host defences and pathogen transmission. Front Cell Infect Microbiol. 2013;3:43. 23971008 10.3389/fcimb.2013.00043 PMC3747359

[5] Francischetti IMB, Sa-Nunes A, Mans BJ, Santos IM, Ribeiro JMC. The role of saliva in tick feeding. Front Biosci-Landmrk. 2009;14:2051–88. 10.2741/3363 PMC2785505 19273185

[6] Abdelwahid E, Rolland S, Teng XC, Conradt B, Hardwick JM, White K. Mitochondrial involvement in cell death of non-mammalian eukaryotes. Bba-Mol Cell Res. 2011;1813:597–607. 10.1016/j.bbamcr.2010.10.008 PMC3033473 20950655

[7] Mao H, Kaufman WR. Profile of the ecdysteroid hormone and its receptor in the salivary gland of the adult female tick, *Amblyomma hebraeum*. Insect Biochem Mol Biol. 1999;29:33–42. 10070743 10.1016/s0965-1748(98)00102-7

[8] L’Amoreaux WJ, Junaid L, Trevidi S. Morphological evidence that salivary gland degeneration in the American dog tick, (Say), involves programmed cell death. Tissue Cell. 2003;35:95–9. 12747931 10.1016/s0040-8166(02)00109-x

[9] Freitas DRJ, Rosa RM, Moura DJ, Seitz AL, Colodel EM, Driemeier D, et al. Cell death during preoviposition period in tick. Vet Parasitol. 2007;144:321–7. 17157985 10.1016/j.vetpar.2006.10.017

[10] Wang H, Zhang XL, Wang X, Zhang BW, Wang MJ, Yang XL, et al. Comprehensive analysis of the global protein changes that occur during salivary gland degeneration in female Ixodid ticks. Front Physiol. 2019;9:1943. 30723423 10.3389/fphys.2018.01943 PMC6349780

[11] Wang Y, Hu S, Tuerdi M, Yu X, Zhang H, Zhou Y, et al. Initiator and executioner caspases in salivary gland apoptosis of *Rhipicephalus haemaphysaloides*. Parasit Vectors. 2020;13:288. 32503655 10.1186/s13071-020-04164-5 PMC7275347

[12] Lu X, Zhang Z, Yuan D, Zhou Y, Cao J, Zhang H, et al. The ecdysteroid receptor regulates salivary gland degeneration through apoptosis in *Rhipicephalus haemaphysaloides*. Parasit Vectors. 2021;14:612. 34930413 10.1186/s13071-021-05052-2 PMC8686549

[13] Wu F, Luo J, Chen Z, Ren QY, Xiao RH, Liu WG, et al. MicroRNA let-7 regulates the expression of ecdysteroid receptor (ECR) in *Hyalomma asiaticum* (Acari: Ixodidae) ticks. Parasite Vector. 2019;12:1–3. 10.1186/s13071-019-3488-6 PMC6521442 31092286

[14] Carrington JC, Ambros V. Role of microRNAs in plant and animal development. Science. 2003;301:336–8. 12869753 10.1126/science.1085242

[15] Smibert P, Lai EC. Lessons from microRNA mutants in worms, flies and mice. Cell Cycle. 2008;7:2500–8. 18719388 10.4161/cc.7.16.6454 PMC2683976

[16] Zhou JL, Zhou YZ, Cao J, Zhang HS, Yu YF. Distinctive microRNA profiles in the salivary glands of related to tick blood-feeding. Exp Appl Acarol. 2013;59:339–49. 22918721 10.1007/s10493-012-9604-3

[17] Liu WG, Luo J, Ren QY, Qu ZQ, Lin HL, Xu XF, et al. A novel miRNA-hlo-miR-2-serves as a regulatory factor that controls molting events by targeting CPR1 in nymphs. Front Microbiol. 2020;11:1098. 32547523 10.3389/fmicb.2020.01098 PMC7274079

[18] Wu F, Luo J, Chen Z, Ren Q, Xiao R, Liu W, et al. MicroRNA let-7 regulates the expression of ecdysteroid receptor (ECR) in *Hyalomma asiaticum* (Acari: Ixodidae) ticks. Parasit Vectors. 2019;12:235. 31092286 10.1186/s13071-019-3488-6 PMC6521442

[19] He J, Kang L. Regulation of insect behavior by non-coding RNAs. Sci China Life Sci. 2024;67:1106–18. 38443665 10.1007/s11427-023-2482-2

[20] Hao J, Luo J, Chen Z, Ren Q, Guo J, Liu X, et al. MicroRNA-275 and its target Vitellogenin-2 are crucial in ovary development and blood digestion of *Haemaphysalis longicornis*. Parasit Vectors. 2017;10:253. 28532427 10.1186/s13071-017-2153-1 PMC5441084

[21] Chawla G, Sokol NS. Hormonal activation of microRNAs via EcR is required for adult morphology and function. Development. 2012;139:1788–97. 22510985 10.1242/dev.077743 PMC3328179

[22] Hackenberg M, Langenberger D, Schwarz A, Erhart J, Kotsyfakis M. In silico target network analysis of de novo-discovered, tick saliva-specific microRNAs reveals important combinatorial effects in their interference with vertebrate host physiology. RNA. 2017;23:1259–69. 28473453 10.1261/rna.061168.117 PMC5513070

[23] Zhou JL, Gong H, Zhou YZ, Xuan XN, Fujisaki K. Identification of a glycine-rich protein from the tick and evaluation of its vaccine potential against tick feeding. Parasitol Res. 2006;100:77–84. 16802136 10.1007/s00436-006-0243-7

[24] Yousef M, Allmer J. miRNomics: microRNA biology and computational analysis. New York: Humana Press; 2014.

[25] Krüger J, Rehmsmeier M. RNAhybrid: microRNA target prediction easy, fast and flexible. Nucleic Acids Res. 2006;34:W451–4. 16845047 10.1093/nar/gkl243 PMC1538877

[26] Rehmsmeier M, Steffen P, Höchsmann M, Giegerich R. Fast and effective prediction of microRNA/target duplexes. RNA. 2004;10:1507–17. 15383676 10.1261/rna.5248604 PMC1370637

[27] Krützfeldt J, Rajewsky N, Braich R, Rajeev KG, Tuschl T, Manoharan M, et al. Silencing of microRNAs with “AntagomiRs.” Nature. 2005;438:685–9. 16258535 10.1038/nature04303

[28] Hu SM, Wang YN, Xu ZM, Zhou YZ, Cao J, Zhang HS, et al. Identification of the Bcl-2 and Bax homologs from and their function in the degeneration of tick salivary glands. Parasite Vector. 2021;14:1. 10.1186/s13071-021-04879-z PMC8336254 34348769

[29] Grabowski JM, Tsetsarkin KA, Long D, Scott DP, Rosenke R, Schwan TG, et al. Flavivirus infection of (black-legged tick) organotypic cultures and applications for disease control. MBio. 2017;8:10. 10.1128/mBio.01255-17 PMC5565970 28830948

[30] de la Fuente J, Kocan KM, Almazan C, Blouin EF. Targeting the tick-pathogen interface for novel control strategies. Front Biosci-Landmrk. 2008;13:6947–56. 10.2741/3201 18508707

[31] Ferreri L, Giacobini M, Bajardi P, Bertolotti L, Bolzoni L, Tagliapietra V, et al. Pattern of tick aggregation on mice: larger than expected distribution tail enhances the spread of tick-borne pathogens. Plos Comput Biol. 2014;10:e1003931. 25393293 10.1371/journal.pcbi.1003931 PMC4230730

[32] Dantas-Torres F, Chomel BB, Otranto D. Ticks and tick-borne diseases: a One Health perspective. Trends Parasitol. 2012;28:437–46. 22902521 10.1016/j.pt.2012.07.003

[33] Tuerdi M, Hu SM, Wang YN, Zhou YZ, Cao J, Zhang HS, et al. Engorgement of ticks blocked by silencing a protein inhibitor of apoptosis. Exp Appl Acarol. 2021;84:623–36. 34136982 10.1007/s10493-021-00637-z

[34] Fahrbach SE, Smagghe G, Velarde RA. Insect nuclear receptors. Annu Rev Entomol. 2012;57:83–106. 22017307 10.1146/annurev-ento-120710-100607

[35] Jiang JH, Ge X, Li ZQ, Wang YQ, Song QS, Stanley DW, et al. MicroRNA-281 regulates the expression of ecdysone receptor (EcR) isoform B in the silkworm. Insect Biochem Mol Biol. 2013;43:692–700. 23707601 10.1016/j.ibmb.2013.05.002

[36] Francis VA, Zorzano A, Teleman AA. dDOR Is an EcR coactivator that forms a feed-forward loop connecting insulin and ecdysone signaling. Curr Biol. 2010;20:1799–808. 20888228 10.1016/j.cub.2010.08.055

[37] Wang M, Wang YH, Chang MM, Wang XL, Shi ZK, Raikhel AS, et al. Ecdysone signaling mediates the trade-off between immunity and reproduction via suppression of amyloids in the mosquito. Plos Pathog. 2022;18:e1010837. 36137163 10.1371/journal.ppat.1010837 PMC9531809

[38] Yang ML, Du BZ, Xu LL, Wang HM, Wang YL, Lin K, et al. Glutamate-GABA imbalance mediated by miR-8–5p and its STTM regulates phase-related behavior of locusts. Proc Natl Acad Sci USA. 2022;120:e2215660120. 36574679 10.1073/pnas.2215660120 PMC9910461

