# Supplementary material for: MicroRNA-34-5p regulates the expression of ecdysteroid receptor (ECR) in the process of salivary gland degeneration of ticks
Source: Parasit Vectors. 2025 May 23;18:187. doi: 10.1186/s13071-025-06842-8 (PMC12100847; doi:10.1186/s13071-025-06842-8)
Supplement: Supplementary file 1 — Additional File 1: Supplementary Table S1. Primers used for quantitative real-time polymerase chain reactions of R. haemaphysaloides RhECR genes and miR-34-5p. Table S2. Primers for R. haemaphysaloides RhECR 3′UTR region and Dual luciferase vector cloning. Table S3. Primers used for overlap extension (SOE) of recombinant PCR to mutant binding sites. Table S4. Primers for RNAi of R. haemaphysaloides RhECR genes. Table S5. The probes for the miRNA and target gene about in situ hybridization. [file 13071_2025_6842_MOESM1_ESM.docx]

**Additional file 1: Table S1.** Primers used for quantitative real-time polymerase chain reactions of *Rhipicephalus haemaphysaloides* *RhECR* genes and mir-34-5p.

| Name | Sequence |
| --- | --- |
| ELF1A-F | CGTCTACAAGATTGGTGGCATT |
| ELF1A-R | CTCAGTGGTCAGGT TGGCAG |
| *RhECR* -F | GGGACCCCAAGAAGAAGAAG |
| *RhECR* -R | GCGTTGTAATGGTAGCCTGATG |
| Stem-mir-34-5p | GTCGTATCCAGTGCAGGGTCCGAGGTATTCGCACTGGATACGACACAACC |
| mir-34-5p -F | GCGTGGCAGTGTGGTTAGCT |
| mir-34-5p -R | AGTGCAGGGTCCGAGGTATT |
| Rhcaspase7-F | GCTTTCGTCGCTTGGGTT |
| Rhcaspase7-R | CGTCGTCCGAGTAGTCTTGCT |

*Abbreviations*: F, forward primer; R, reverse primer. mir-34-5p uses stem-loop method to design primers, and Stem-mir-34-5p is the primer used in reverse transcription.

**Additional file 1: Table S2.** Primers for *Rhipicephalus haemaphysaloides* *RhECR* 3′‑UTR region and Dual luciferase vector cloning.

| Name | Sequence |
| --- | --- |
| 3′‑UTR -GSP-RhECR-R1 | CCTTGGGCAACATGAACGCCGAGATGTG |
| 3′‑UTR -GSP-RhECR-R2 | TCGCTCAAGGTGCAGAACAAGAAGCTGC |
| pmir-GLO-RhECR-F | tgtaattctagttgtttaaacTAGTGGTGGGGGGAGGGGT |
| pmir-GLO-RhECR-R | caggtcgactctagactcgagGCAACCACTCCCCTCAAGAACTTC |

*Abbreviations*: F, forward primer; R, reverse primer.

**Additional file 1: Table S3.** Primers used for overlap extension (SOE) of recombinant PCR to delete BH domain.

| Name | Sequence |
| --- | --- |
| RhECR-mutant-F | TGGGCCAGCAGCACGCACAGAGGAGCTTCAGGTCACGTTTTT |
| RhECR-mutant-R | GACCTGAAGCTCCTCTGTGCGTGCTGCTGGCC |

*Abbreviations*: F, forward primer; R, reverse primer.

**Additional file 1: Table S4.** Primers for RNAi of *Rhipicephalus haemaphysaloides* *RhBcl-2* and *RhBax* genes.

| Name | Sequence |
| --- | --- |
| *RhECR* dsRNA-S1 | GGATCCTAATACGACTCACTATAGG CAGCACAACACGAGAGAGC |
| *RhECR* dsRNA-A1 | GCTGGTTGTTGGCAAACACGATAGAA |
| *RhECR* dsRNA-S2 | CAGCACAACACGAGAGAGC |
| *RhECR* dsRNA-A2 | GGATCCTAATACGACTCACTATAGG GCTGGTTGTTGGCAAACACGATAGAA |

*Abbreviations*: S, forward primer; A, reverse primer, the sequence in underlined indicates the sequence of the T7 promoter.

**Additional file 1:** **Table S5.** **The probes for the miRNA and target gene about *in situ hybridization***

| Name | Sequence |
| --- | --- |
| *RhECR* probes | FAM-GGCTCTGCTGTGGACTGCCTGCAAGTGGCC-FAM |
| mir-34-5p probes | CY3-TGGCAGTGTGGTTAGCTGGTTGT |
|  |  |
